# Role of *Acorus calamus* in preventing depression, anxiety, and oxidative stress in long-term socially isolated rats

**DOI:** 10.14202/vetworld.2023.1755-1764

**Published:** 2023-08-28

**Authors:** Ashwin Rohan Rai, Teresa Joy, Meghana Poojari, Mangala M. Pai, Amit Massand, B. V. Murlimanju

**Affiliations:** 1 Department of Anatomy, Kasturba Medical College, Mangalore, Manipal Academy of Higher Education, Manipal, India; 2 Department of Anatomy, American University of Antigua College of Medicine, University Park, Jabberwock Beach Road, Coolidge, St. John’s, Antigua, West Indies; 3 Department of Anatomy, Basaveshwara Medical College and Hospital, Chitradurga, India

**Keywords:** antidepressant, antioxidative effects, brain tissue, coronavirus disease 2019 pandemic

## Abstract

**Background and Aim:**

Social isolation stress (SIS) and individual housing have been shown to cause abnormal cognitive insufficiencies, altered anxiety levels, and signs of psychiatric diseases. *Acorus calamus* (AC), commonly known as Sweet Flag, has been widely used in India to treat neurological, metabolic, and respiratory disorders, indicating its potential therapeutic value. This study aimed to determine the antidepressant and antioxidative effects of AC on rats subjected to long-term, social isolation-induced stress.

**Materials and Methods:**

This study involved 2-month-old male rats (24) weighing approximately 180–200 g bred in-house. The rats were divided into four groups (n = 6): Group 1 received saline, Group 2 received SIS, Group 3 received only 50 mg/kg AC, and Group 4 received 50 mg/kg AC and SIS for 6 weeks. After this, behavioral, biochemical, and neuronal assays were conducted.

**Results:**

Behavioral experiments showed significantly higher activity levels (p < 0.001) in AC-treated rats than in the SIS group. In addition, rats subjected to SIS with AC treatment exhibited enhanced total antioxidants, superoxide dismutase, and neuronal assays compared to rats subjected to SIS alone.

**Conclusion:**

*Acorus calamu*s treatment improved the antidepressant and antioxidant potential against SIS in rat brain tissue. Moreover, we proved that AC can effectively reverse the neurotoxicity induced by SIS in animal models. As we battle against the coronavirus disease 2019 pandemic and social isolation, AC could be considered a supplementary treatment to alleviate depressive-like symptoms in our present-day lifestyle.

## Introduction

Social isolation (individual housing) is a complete lack of contact between members of the same order [1, 2]. It is a psychosocial stressor [3] that can lead to alterations in social behavior [4], neurochemical factors [5], and specific anatomical structures [6], as well as symptoms such as compulsive-neurotic behavior, sleeplessness, digestion problems, and depression [7], in both animals and humans. During the coronavirus disease 2019 (COVID-19) pandemic, social isolation was crucial in limiting the spread of the virus, and recent research by “The Lancet” medical journal [8] states that a minimum of 10 days of isolation can result in long-term psychiatric symptoms. In a rat model, adolescent social isolation was shown to be a powerful stressor that reliably mimics stress in humans [9]. Researchers have also investigated the consequences of combining maternal separation during preweaning with social isolation during postweaning [10]. Walker *et al*. [11] found that social isolation during preadolescence and late adolescence can enhance anxiety-related behaviors. In addition, Zorzo *et al*. [12] reported that socially isolated rodents from preadolescence to mid-adolescence are likely to exhibit increased anxiety-related behavior. However, different experimental studies have varied regarding the onset, duration, age, and sex of rats subjected to social isolation stress (SIS).

Early childhood SIS, whether acute or chronic, has been linked to abnormal cognitive functions [13, 14], altered anxiety levels [15], and various psychiatric disease symptoms [16]. Rats subjected to SIS have shown symptoms similar to those of humans, including forgetfulness, depression, anxiety, and schizophrenia-related disorders [17, 18]. Several studies on rodents have demonstrated that a lack of social interaction can lead to lifelong behavioral abnormalities and intellectual deficits, including memory impairment, challenges with focus and learning, and poor managerial or governing skills [2, 5, 19]. Depression is another disorder observed after chronic SIS [20]. Experimental models of socially isolated adolescent and adult rats have displayed behavioral alterations associated with oxidative stress (OS) [21], including neurobiological changes in the brain that affect brain activity [22–27]. Social isolation and loneliness often coexist and are interrelated. Although the literature offers clear insight into the risks associated with social isolation, therapeutics based on social cognition and behavioral principles have thus far shown unsatisfactory outcomes [28].

The antioxidant defense mechanism of the central nervous system is sufficient to counteract continuous oxidative damage despite being sensitive to OS. Oxidative stress has been extensively studied in neurological diseases using various stressors. Stress-induced oxidative damage in the brain can alter cellular homeostasis. Rai *et al*. [29] demonstrated stress-induced memory and learning impairments associated with damage to the antioxidant system of the brain. In addition, SIS-induced for 8 weeks has been shown to cause an antioxidant imbalance, leading to reduced release of reactive oxygen species (ROS) and antioxidant enzymes such as superoxide dismutase (SOD), catalase, and glutathione peroxidase (GPx) in the rat brain [21].

Early SIS has been linked to impaired mitochondrial function, leading to increased nitric oxide (NO) and ROS production and a significant decrease in glutathione and ATP levels [30, 31]. Researchers have confirmed that the nitrenergic system and N-methyl-D-aspartate (NMDA) receptors promote depression-like behaviors and have a proconvulsant effect in adult mice [32, 33]. Studies involving limbic areas have reported that SIS induces elevated levels of NO and increased expression of NO synthase isoforms [34, 35].

Social isolation stress has been found to significantly affect the prefrontal cortex [36]. Tang *et al*. [37] found that the hippocampal and prefrontal sequences interact at various time points during memory-guided decision-making. Recent research indicates that the medial prefrontal cortex (mPFC) is crucial in regulating various cognitive functions, including attention, habit formation, working memory, spatial memory, and long-term memory. The mPFC also controls cognitive domains and stimuli top-down, filtering data based on similarity while integrating them over time [38, 39]. In other words, activity in the mPFC can be influenced by the history of a stimulus, which can affect attention modulation [40]. The atrophy and cell death of neurons in the mPFC subjected to stress significantly influence cognitive and emotional processes [41]. Social isolation rearing has been shown to affect prefrontal cortex activation, resulting in aggressive behavior in rats [42]. Clinical evidence has linked mPFC dysfunction to depression [43] and post-traumatic stress disorder [44]. Hence, investigating morphological changes in neurons in these areas could provide additional insights that can be correlated with OS and behavioral function in the rat brain.

*Acorus calamus* (AC) Linn. (*Acoraceae*), known as Vacha in Sanskrit and Sweet Flag in English, is an annual, semiaquatic, aromatic herb widely used in Ayurveda (as part of conventional Indian treatment) and traditional Chinese medicine [45]. This plant, which can grow up to 6 feet tall, is found in most regions of Asia, Europe, and North America and has long, slender leaves, flowers, fruits, and rhizomes. The entire plant has medicinal value, with rhizomes commonly used in India to treat disorders such as seizures, insanity, asthma, diarrhea, insomnia, and hysteria [46]. The rhizome and leaves of AC contain several phytochemical compounds, including phenylpropanoids, sesquiterpenoids, and monoterpenes, with α and β-asarone and eugenol being predominantly present in the rhizome. Vacha is commonly used in Ayurveda to manage various health issues affecting different body systems, including the respiratory, neurological, gastrointestinal, metabolic, liver, and kidney systems. The rhizome possesses antidepressant activities [47] and is a curative agent for attention deficit disorders and memory loss [48]. *Acorus calamus* has numerous health benefits, including neuroprotection, antioxidant properties, antidepressant effects, anticonvulsant activity, antihypertensive properties, anti-inflammatory effects, immunomodulatory activity, cardioprotective effects, and potential benefits for obesity [45, 49]. At the start of the COVID-19 pandemic, social isolation became one of the major remedial measures. Therefore, we aimed to investigate the role of AC in reversing the psychological effects of long-term SIS at the neuromorphological, behavioral, and biochemical levels.

Research has shown that AC exhibits a neuroprotective effect in autism-induced Wistar rats [50]. In addition, the administration of AC has been found to be beneficial in treating epilepsy [51, 52] and preventing the formation of FeCl-induced epileptogenesis by modulating antioxidant enzymes [53]. *Acorus calamus* has also shown effectiveness against noise stress-induced memory impairment [54] and has been proven to prevent OS, memory loss, and anxiety due to lipopolysaccharide-induced neuroinflammation [48]. *Acorus calamus* rhizome powder has demonstrated a beneficial effect on restraint stress-induced modulatory changes in Na-K-ATPase activity, antioxidants, and cognitive functions [55]. Furthermore, AC rhizome extract has shown effectiveness in alleviating depression symptoms, making it a valuable supplement for individuals seeking treatment [56]. Forouzanfar and Hosseinzadeh [57] argued that AC could improve the behavioral and biochemical changes induced by painful environmental neuropathy through its antioxidant and anti-inflammatory effects.

This study aimed to evaluate the following: (1) behavioral changes (motor and cognitive functions) in rats subjected to SIS and AC-treated rat brains; (2) the level of antioxidants in rat brains subjected to SIS and AC-treated rats; (3) histomorphological changes in the neurons of the mPFC in rats subjected to SIS AC treatment; and (4) the effect of AC on oxidative damage, as well as behavioral and histomorphological changes in rat brains subjected to SIS.

## Materials and Methods

### Ethical approval

The study was approved by Institutional Animal Ethics Committee of Kasturba Medical College, Mangalore (KMC/MNG/IAEC/10-2019).

### Study period and location

This study was conducted from July 2020 to December 2020 at Central Animal House, Kasturba Medical College, Mangalore.

### Animals

This study used 2-month-old male rats (weighing 180 g ± 20) bred in-house. Food and water were available to animals *ad libitum*. They were retained in controlled environments with respect to humidity, temperature, and light and dark cycles. Paddy husk was used as bedding and rats were housed in polypropylene cages. The rules set forth by the Indian Government for the use of laboratory animals were followed in this investigation [58].

*Acorus calamus* powder was purchased from Natural Remedies, Bangalore, India. The experiment included the following animal groups, each consisting of six rats (n = 6). The initial weight of all rats was recorded before the experiment commenced. The experimental rats received AC orally through an oral gavage tube. The rats were allowed to acclimatize for a week before being randomly divided.

- Group 1: Six socially grouped (SG) rats received saline (3 rats/cage)
- Group 2: Six rats subjected to SIS for 6 weeks (1 rat/cage)
- Group 3: Six rats received only 50 mg/kg of AC daily for 6 weeks (3 rats/cage)
- Group 4: Six rats received AC at 50 mg/kg daily + SIS for 6 weeks (1 rat/cage).

### Drug design

*Acorus calamus* powder was administered at 50 mg/kg daily [45]. This administration was done orally through a feeding cannula with water as a vehicle. *Acorus calamus* powder was purchased from Natural Remedies, Bangalore, India. All other chemicals and reagents were high-performance liquid chromatography or analytical grade (Sigma, St. Louis, MO, U.S.A.) purchased from Sri Durga Laboratories, Mangalore, Karnataka, India.

### Behavioral tests

#### Open-field test

The open-field test is the most commonly employed method for analyzing rats’ emotional, exploratory, and motor activities [59]. A rectangular open-field box (Techno, Lucknow, India) measuring 100 × 100 × 40 cm with 25 squares (5 × 5 cm) marked on the floor was used. The apparatus was uniformly illuminated with a 60-watt bulb positioned 60 cm above the center of the field. Each rat was placed in a corner of the chamber, and the total time spent in the peripheral and central squares was recorded. The total time provided for exploration in each session was 5 min. Rearing and grooming behaviors were also assessed as the most reliable indicators of emotionality in rodents. Less time spent in the central squares than the peripheral squares reflect avoidance of anxiogenic places, while the number of squares crossed indicates exploratory behavior. Grooming behavior is involved in maintaining body hygiene, caring for the body surface, thermoregulation, and reducing stress. This test was conducted after 6 weeks of SIS.

### Tail suspension test (TST)

The TST was performed as defined by Nandi *et al*. [60]. Using adhesive tape, each rat was suspended from the tip of the tail on a horizontal bar 50 cm above the benchtop in a visibly isolated area. The test duration was 6 min. It was video-recorded by an experimenter blinded to the experimental group of rats to measure the total mobility time. The TST is a tool used to assess depressive- and anxiety-like behavior.

### Passive avoidance test

The memory recall of the rats in all groups was tested using a passive avoidance device, as described and applied in a study by Rai *et al*. [29]. This test evaluates a rat’s capacity to recall a foot shock administered 24 h before the test. The passive avoidance device is a box of wood with one big, bright compartment and one small, dark compartment with a grid floor connected to a shock resource. The experiment involved three components:

- Exploration test: On the 1^st^ day of testing, the rats were positioned for exploration in the center of the bright compartment, facing away from the entry to the small, dark compartment. The door separating both compartments was kept open, and three test trials lasting 5 min each were conducted after the rats had 5 min to explore each chamber. The time spent in each compartment was noted during every trial, and the rats were placed in the home cage after each trial.
- Aversive stimulation and learning phase (passive avoidance acquisition): After the third test trial, a foot shock (50 Hz, 1.5 mA, 1 s) was applied across the grid floor immediately after the rats entered the dark compartment. The rats were given an additional 10 s to establish a link between the characteristics of the chamber and the foot shock. The rats were then placed back into the home cage.
- Retention test: The memory retention test was conducted 24 h after the foot shock. The rats were placed in the bright compartment, and the time taken (latency) to enter the dark compartment for the 1^st^ time was recorded. The rats were given a maximum of 300 s to explore. Normal rats do not explore the dark compartment, where they received a shock the preceding day, restricting their natural behavior. A decreased latency to enter the dark section suggests inadequate memory retention.

### Biochemical study

After 24 h, biochemical tests were conducted. The rats were sacrificed through cervical decapitation, and the entire brain was quickly separated and cleaned with 0.1 M/L saline phosphate buffer (pH 7.4). The tissue was then weighed and homogenized (1:10 w/v) in 0.1 M/L saline phosphate buffer. After centrifuging the homogenate at 10,000× *g* for 20 min at 4°C, a portion of the supernatant was removed for biochemical analysis.

### Estimation of total antioxidants (TAO)

The assay principle involves a Fenton-type reaction, in which a standardized solution of the Fe-Ethylenediaminetetraacetic acid complex combines with hydrogen peroxide to produce hydroxyl radicals. These ROS break down benzoate, generating compounds that react with thiobarbituric acid. Antioxidants in the brain homogenate inhibit the production of thiobarbituric acid–reactive substances. The reaction was quantified using spectrophotometry, and the inhibition rate of color development was proportional to the concentration of antioxidant activity. The TAO level was calculated following the method described by Koracevic *et al*. [61].

### Assay of SOD activity

The procedure developed by Marklund and Marklund [62] and Rao *et al*. [63] was used to measure SOD activity. The reaction was conducted in a solution containing 5.6 × 10^−5^ M nitroblue tetrazolium, 1.17 × 10^−6^ M riboflavin, and 1 × 10^−2^ M methionine in 0.05 M potassium phosphate buffer (pH 7.8), along with appropriately diluted tissue homogenate in a volume of 3 mL. The solution was placed in a beaker and illuminated for 10 min using a 15-volt fluorescent bulb inside an aluminum-lined foil box. The control sample was prepared without the enzyme. The absorbance was determined at 560 nm using a Systronic-117 ultraviolet-visible spectrophotometer, and SOD activity was quantified as the specific activity of the enzyme (U/mg protein). The technique developed by Lowry *et al*. [64] was used to determine the protein content.

### Histomorphological studies

**Figure-1:**
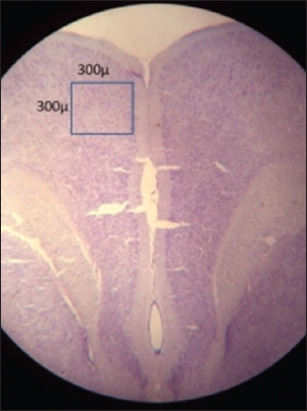
Medial prefrontal cortex stained (Cresyl violet stain).

Transcardiac perfusion with 10% formalin and 0.9% saline was conducted after the rats were deeply anesthetized with ether. The rats were decapitated, and their brains were preserved for 48 h in 10% formalin. Coronal sections of the prefrontal cortex with a thickness of 4–6 μm were cut using a rotary microtome (Jung Biocutt 2035, Leica, Germany). Afterward, 25 sections were serially mounted on air-dried gelatinized slides from each animal. Cresyl violet stain was used to stain the sections [65]. The Nissl substance was stained with cresyl violet and appeared granular purple-blue in the cytoplasm of neurons. High-quality images were taken with an Olympus digital camera (DP75) connected to an Olympus microscope (Japan) with 20× objectives. NIS Eléments Br Version 4.30 software (Nikon Co., India) was used to count the number of neurons in each image, and healthy neurons in a 300 μ^2^ area of the mPFC were counted (Figure-1). Six sections from each rat were considered, and the count excluded cells that were deeply stained, shrunken, or had fragmented nuclei. The slides from various rat groups were decoded to prevent bias when counting the cells manually.

### Statistical analysis

The data were expressed as mean ± SE, and a one-way analysis of variance was performed to assess the significance of the differences among the groups. A significance level of p < 0.05 was considered significant.

## Results

### Open-field test

#### Rearing and grooming

**Figure-2:**
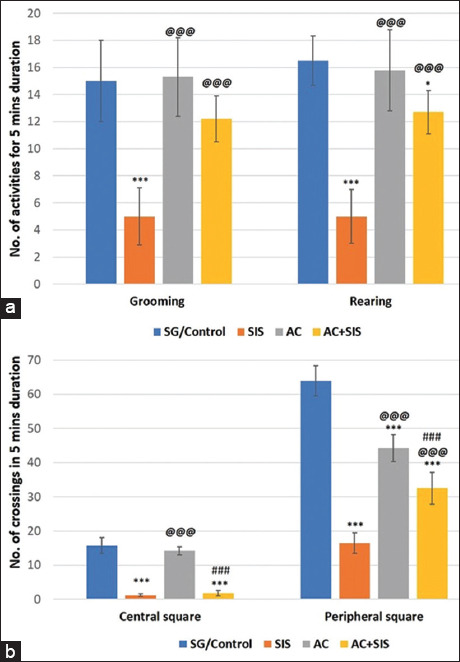
(a) Comparison of open field activity - grooming and rearing activity in rats of different group. Values are expressed as mean ± SD (n = 6); Grooming - SG/Control versus SIS is *** = p < 0.001, SIS versus AC is @@@ = p < 0.001, SIS versus AC+SIS is @@@ = p < 0.001. Rearing – SG/Control versus SIS is *** = p < 0.001, SG/Control versus AC+SIS is * = p < 0.05, SIS versus AC is @@@ = p < 0.001, SIS versus AC+SIS is @@@ = p < 0.001. (b) Comparison of Open field activity- central and peripheral square crossing in rats of different group. Values are expressed as mean ± SD (n = 6); Central square crossing - SG/Control versus SIS is *** = p < 0.001, SG/Control versus AC+ SIS is *** = p < 0.001, SIS versus AC is @@@ = p < 0.001, AC versus AC+SIS is ### = p < 0.001. Peripheral square crossing – SG/Control versus SIS is *** = p < 0.001, SG/Control versus AC is *** = p < 0.001, SG/Control versus AC+SIS is *** = p < 0.001, SIS versus AC is @@@ = p < 0.001, SIS versus AC+SIS is @@@ = p < 0.001, AC versus AC+SIS is ### = p < 0.001. SIS=Social isolation stress, AC=*Acorus calamus*.

Rearing and grooming activities, indicative of emotional behavior, were significantly reduced (p < 0.001) in socially isolated rats compared to SG rats. However, the rats subjected to AC treatment with SIS showed an increase in these emotional activities compared to the SIS group. *Acorus calamus*-treated rats also showed a significant increase (p < 0.001) in these activities when compared to SIS rats (Figure-2a).

#### Number of crossings in a central square

Social isolation stress rats demonstrated significantly reduced central square crossings (p < 0.001), indicating anxiety-like behavior. *Acorus calamus* treatment in SIS rats was unable to completely reverse the anxiety-like behavior (p > 0.01) in the open-field test compared to SG rats (Figure-2b).

#### Number of crossings in a peripheral square

The number of peripheral square crossings was significantly reduced (p < 0.001) in SIS rats compared to SG rats, indicating an impact on general locomotor activity. *Acorus calamus* treatment in SIS rats significantly enhanced (p < 0.001) the number of crossings in peripheral squares compared to SIS rats, suggesting enhanced locomotor and exploratory activities. *Acorus calamus* treatment in SIS rats also resulted in a significant decrease (p < 0.001) in peripheral square crossings compared to the AC treatment group (Figure-2b).

#### Tail suspension test

**Figure-3:**
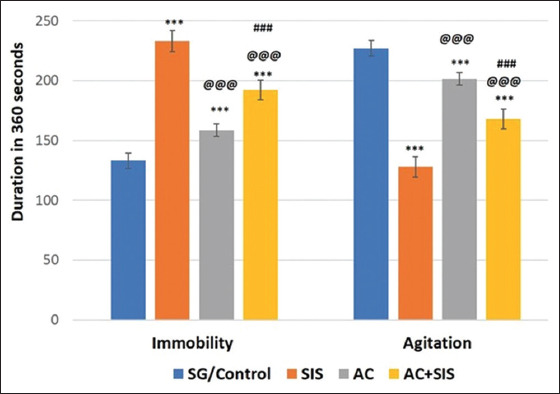
Comparison of tail suspension test in rats of different group. Values are expressed as mean ± SD (n = 6); Immobility time - SG/Control versus SIS is *** = p < 0.001, SG/Control versus AC is *** = p < 0.001, SG/Control versus AC+ SIS is *** = p < 0.001, SIS versus AC is @@@ = p < 0.001, SIS versus AC+SIS is @@@ = p < 0.001, AC versus AC+SIS is ### = p < 0.001. Agitation time – SG/Control versus SIS is *** = p < 0.001, SG/Control versus AC is *** = p < 0.001, SG/Control versus AC+SIS is *** = p < 0.001, SIS versus AC is @@@ = p < 0.001, SIS versus AC+SIS is @@@ = p < 0.001, AC versus AC+SIS is ### = p < 0.001. SIS=Social isolation stress, AC=*Acorus calamus*.

The immobility time was significantly higher (p < 0.001) in SIS rats than SG rats, indicating depressive-like behavior. However, the SIS rats treated with AC showed a reduced immobility time compared to the SIS rats, indicating a reversal of depressive-like behavior (Figure-3).

#### Passive avoidance test

**Figure-4:**
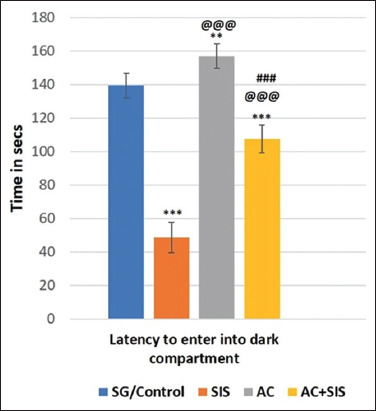
Comparison of passive avoidance test in rats of different group. Values are expressed as mean ± SD (n = 6); Latency to enter into dark compartment - SG/Control versus SIS is *** = p < 0.001, SG/Control versus AC is ** = p < 0.01, SG/Control versus AC+SIS is *** = p < 0.001, SIS versus AC is @@@ = p < 0.001, SIS versus AC+SIS is @@@ = p < 0.001, AC versus AC+SIS is ### = p < 0.001. SIS=Social isolation stress, AC=*Acorus calamus*.

The time taken for SIS rats to enter the dark compartment was significantly shorter (p < 0.001) than SG rats, suggesting poor retrieval of learning behavior. Treatment with AC alone enhanced the time of entry into the dark compartment, indicating improved learning abilities. However, AC treatment in SIS rats did not increase the duration to enter the dark compartment, as the potential to enter the dark compartment was significantly lower (p < 0.001) compared to SG rats. This result showed that SIS rats treated with AC had poor recovery of learning behavior but had an enhanced latency to enter the dark compartment compared to rats subjected to isolated stress alone (Figure-4).

#### Estimation of TAOs

**Figure-5:**
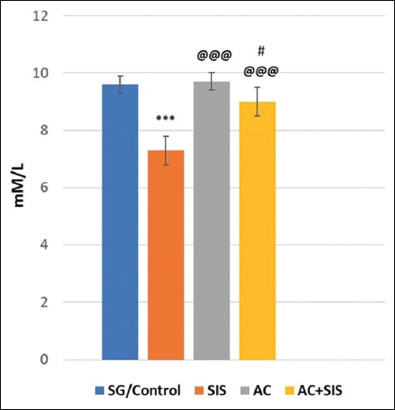
Comparison of TAO level in rats of different group. Values are expressed as mean ± SD (n = 6); SG/Control versus SIS is *** = p < 0.001, SIS versus AC is @@@ = p < 0.001, SIS versus AC+SIS is @@@ = p < 0.001, AC versus AC+SIS is # = p < 0.05. TAO=Total antioxidants, SIS=Social isolation stress, AC=*Acorus calamus*.

The TAO levels in the brain homogenate were significantly reduced (p < 0.001) in isolated rats compared to SG rats. However, AC treatment in SIS rats resulted in a significant enhancement (p < 0.001) in the level of TAO in the brain tissue compared to SIS rats. Rats subjected to AC treatment alone also showed a highly significant (p < 0.001) increase in brain TAO levels when compared to SIS rats (Figure-5).

#### Assay of SOD activity

**Figure-6:**
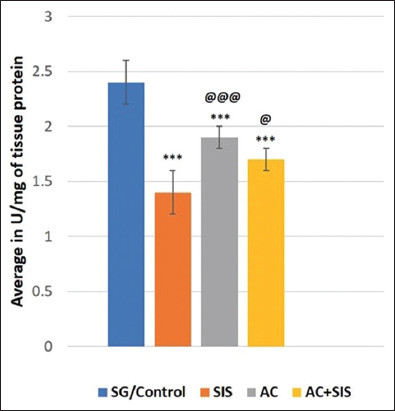
Comparison of superoxide dismutase in rats of different group. Values are expressed as mean ± SD (n = 6); SG/Control versus SIS is *** = p < 0.001, SG/Control versus AC is *** = p < 0.001, SG/Control versus AC+ SIS is *** = p < 0.001, SIS versus AC is @@@ = p < 0.001, SIS versus AC+SIS is @ = p < 0.05. SIS=Social isolation stress, AC=*Acorus calamus*.

Social isolation stress rats exhibited a significant reduction (p < 0.001) in the level of SOD in the brain tissue compared to SG rats. However, AC-treated SIS rats revealed a significant increase (p < 0.05) in the level of SOD in the brain homogenate compared to SIS and SG rats (p < 0.001). *Acorus calamus* treatment alone showed a significant (p < 0.001) increase in brain TAO levels compared to SG and SIS rats (Figure-6).

#### Neuronal assay of the mPFC

**Figure-7:**
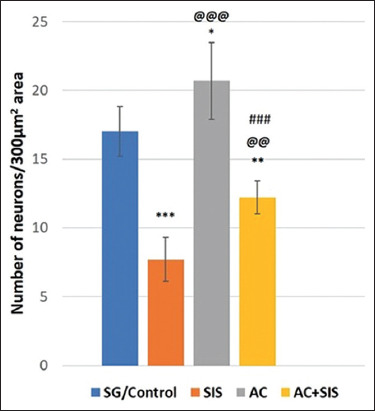
Comparison of neurons in medial pre-frontal cortex in rats of different group. Values are expressed as mean ± SD (n = 6); SG/Control versus SIS is *** = p < 0.001, SG/Control versus AC is * = p < 0.05, SG/Control versus AC+SIS is ** = p < 0.01, SIS versus AC is @@@ = p < 0.001, SIS versus AC+SIS is @@ = p < 0.01, AC versus AC+SIS is ### = p < 0.001. SIS=Social isolation stress, AC=*Acorus calamus*.

Social isolation stress rats exhibited significantly fewer normal neurons compared to the SG (p < 0.001) and AC treatment groups (p < 0.05), suggesting degenerating neurons. *Acorus calamus* treatment in SIS rats resulted in a significant increase in normal neurons compared to SIS rats (p < 0.01) and AC-treated rats (p < 0.001) (Figure-7).

## Discussion

### Observation of anxiety and emotional, locomotor, and exploratory activities in rats subjected to SIS and AC treatment

In this study, we observed that SIS rats spent less time in the center squares, displaying increased anxiety-like behavior than SG rats, which was not reversed by AC treatment. However, the locomotor and exploratory activities of the SIS rats were lower in the peripheral squares compared to the SG rats, and this impairment was significantly improved by AC treatment. These findings are consistent with Grigoryan *et al*. [66], who evaluated behavioral alterations and brain molecular changes in SIS mice. The open-field test is the most reliable indicator of emotionality in rodents. Self-grooming is an important behavior observed in rodents, primarily to maintain body hygiene, thermoregulation, chemocommunication, and stress reduction [67]. Chronic restraint stress, which has been well documented [68], is known to disrupt emotional activities, aligning with the findings of our study that emotional activities are significantly reduced in SIS rats compared to SG rats. In this study, AC treatment enhanced emotional activities (grooming and rearing), which were suppressed by SIS, consistent with Rauniar *et al*. [69]. In an acute oral toxicity test, raw and purified AC at doses of up to 2000 mg/kg did not cause adverse effects when administered for 14 d [70]. In addition, the SIS rats showed a significantly higher immobility time in the TST than the SG rats, indicating anxiety- and depressive-like behavior, as demonstrated in Grigoryan *et al*. [66]. However, AC treatment reversed this behavior [71, 72].

### Observation of learning abilities and memory retention in rats subjected to SIS and AC treatment

Social isolation stress rats exhibit symptoms similar to human anxiety, depression, schizophrenia, and memory loss [17, 18, 73]. Our findings showed that the SIS rats displayed poor recovery of learning behavior in the passive avoidance test compared to the SG rats, which was improved by the antioxidant and anti-inflammatory effects of AC treatment. This result is consistent with a previous study that evaluated the potential of AC [68].

### Observation of antioxidant molecules and enzymes in rat brain homogenate after being subjected to SIS and AC treatment

Social isolation stress-induced for 8 weeks caused an antioxidant imbalance, inducing reduced production of ROS and decreased levels of SOD, catalase, and GPx in the rat brain [66, 74]. Our study confirmed that SIS caused oxidative damage in the rat brain, with significantly lower SOD levels and TAO activity compared to SG rats. However, AC treatment in the SIS rats led to a highly significant increase in TAO activity and a slight increase in SOD activity, verifying the antioxidant potential of AC. Shukla *et al*. [75] also demonstrated the efficacy of AC in increasing SOD activity in rats with ischemia induced by middle cerebral artery obstruction.

### Changes in neuronal numbers in the mPFC of rats subjected to SIS and AC treatment

**Figure-8:**
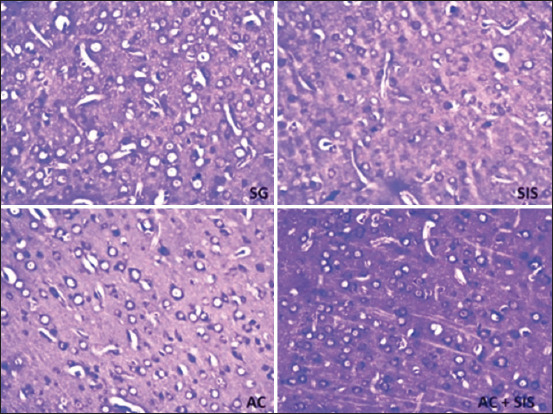
Medial prefrontal cortex – under 20×, SG versus others.

The mPFC is crucial in integrating cognitively and emotionally significant information [39, 40]. Social isolation stress significantly affects the prefrontal cortex of the brain [36]. The atrophy and cell death of neurons in mPFCs subjected to stress significantly influence cognitive and emotional processes [41]. Social isolation stress resulted in a loss of neurons associated with declining retrieval memory in the mPFC compared to SG rats, which was reversed by AC treatment (Figure-8). Currently, there is limited data on AC’s role in reversing neuronal degeneration in the mPFC.

## Conclusion

The rat model of SIS used in this study effectively mimics human stress conditions. The long-term effect of stress on neuronal loss, including cognitive decline and accelerated aging, is well known. Thus, we focused on the role of antioxidants, cognitive behavior, and neuronal assays in the mPFC. *Acorus calamus*, the promising drug in this study, exhibited antidepressant and antioxidant potential against SIS in brain tissue. The results indicate that AC effectively combats SIS-induced neurotoxicity in animal models. Considering the importance of social therapy during the COVID-19 pandemic, AC could serve as a valuable supplement in combating SIS-induced psychiatric symptoms and in addressing the challenges of our present-day lifestyle.

## Authors’ Contributions

ARR and TJ: Planned and conducted the study and drafted the manuscript. MP and AM: Conducted the animal experiment and stained the slides. MMP and BVM: Statistical analysis, prepared graphs, and drafted and revised the manuscript All authors have read, reviewed, and approved the final manuscript.

## References

[1] Arakawa H (2018). Ethological approach to social isolation effects in behavioral studies of laboratory rodents. Behav. Brain Res.

[2] Mumtaz F, Khan M.I, Zubair M, Dehpour A.R (2018). Neurobiology and consequences of social isolation stress in animal model-A comprehensive review. Biomed. Pharmacother.

[3] Brandt L, Liu S, Heim C, Heinz A (2022). The effects of social isolation stress and discrimination on mental health. Transl. Psychiatry.

[4] Cacioppo J.T, Cacioppo S (2018). The growing problem of loneliness. Lancet.

[5] Vitale E.M, Smith A.S (2022). Neurobiology of loneliness, isolation, and loss:Integrating human and animal perspectives. Front. Behav. Neurosci.

[6] Bzdok D, Dunbar R.I.M (2022). Social isolation and the brain in the pandemic era. Nat. Hum. Behav.

[7] Rogers J.P, Chesney E, Oliver D, Pollak T.A, McGuire P, Fusar-Poli P, Zandi M.S, Lewis G, David A.S (2020). Psychiatric and neuropsychiatric presentations associated with severe coronavirus infections:A systematic review and meta-analysis with comparison to the COVID-19 pandemic. Lancet Psychiatry.

[8] Brooks S.K, Webster R.K, Smith L.E, Woodland L, Wessely S, Greenberg N, Rubin G.J (2020). The psychological impact of quarantine and how to reduce it:Rapid review of the evidence. Lancet.

[9] Rivera-Irizarry J.K, Skelly M.J, Pleil K.E (2020). Social isolation stress in adolescence, but not adulthood, produces hypersocial behavior in adult male and female C57BL/6J mice. Front. Behav. Neurosci.

[10] Weiss I.C, Domeney A.M, Moreau J.L, Russig H, Feldon J (2001). Dissociation between the effects of pre-weaning and/or post-weaning social isolation on prepulse inhibition and latent inhibition in adult Sprague-Dawley rats. Behav. Brain Res.

[11] Walker D.M, Cunningham A.M, Gregory J.K, Nestler E.J (2019). Long-term behavioral effects of post-weaning social isolation in males and females. Front. Behav. Neurosci.

[12] Zorzo C, Méndez-López M, Méndez M, Arias J.L (2019). Adult social isolation leads to anxiety and spatial memory impairment:Brain activity pattern of COx and c-Fos. Behav. Brain Res.

[13] Ren Y, Savadlou A, Park S, Siska P, Epp J.R, Sargin D (2023). The impact of loneliness and social isolation on the development of cognitive decline and Alzheimer's disease. Front. Neuroendocrinol.

[14] Cardona M, Andrés P (2023). Are social isolation and loneliness associated with cognitive decline in ageing?. Front. Aging Neurosci.

[15] Hwang Y, Massimo L, Aryal S, Hodgson N.A (2022). The relationship between social isolation and anxiety in people with cognitive impairment in the United States. Int. J. Geriatr. Psychiatry.

[16] Famitafreshi H, Karimian M (2018). Social isolation rearing induces neuropsychiatric diseases:Updated overview. Mol. Neuropsychiatry.

[17] Mann F, Wang J, Pearce E, Ma R, Schlief M, Lloyd-Evans B, Sarah I, Johnson S (2022). Loneliness and the onset of new mental health problems in the general population. Soc. Psychiatry Psychiatr. Epidemiol.

[18] Read S, Comas-Herrera A, Grundy E (2020). Social isolation and memory decline in later-life. J. Gerontol. B Psychol. Sci. Soc. Sci.

[19] Bianchi M, Fone K.F.C, Azmi N, Heidbreder C.A, Hagan J.J, Marsden C.A (2006). Isolation rearing induces recognition memory deficits accompanied by cytoskeletal alterations in rat hippocampus. Eur. J. Neurosci.

[20] Zhao X, Sun L, Jia H, Meng Q, Wu S, Li N, He S (2009). Isolation rearing induces social and emotional function abnormalities and alters glutamate and neurodevelopment-related gene expression in rats. Prog. Neuropsychopharmacol. Biol. Psychiatry.

[21] Cho J.H.J, Olmstead R, Choi H, Carrillo C, Seeman T.E, Irwin M.R (2019). Associations of objective versus subjective social isolation with sleep disturbance, depression, and fatigue in community-dwelling older adults. Aging Ment. Health.

[22] Grigoryan G.A, Pavlova I.V, Zaichenko M.I (2022). Effects of social isolation on the development of anxiety and depression-like behavior in model experiments in animals. Neurosci. Behav. Physiol.

[23] Lapiz M.D.S, Fulford A, Muchimapura S, Mason R, Parker T, Marsden C.A (2003). Influence of postweaning social isolation in the rat on brain development, conditioned behavior, and neurotransmission. Neurosci. Behav. Physiol.

[24] Westenbroek C, Den Boer J.A, Veenhuis M, Ter Horst G.J (2004). Chronic stress and social housing differentially affect neurogenesis in male and female rats. Brain Res. Bull.

[25] Schoenfeld T.J, Gould E (2012). Stress, stress hormones, and adult neurogenesis. Exp. Neurol.

[26] Liu C, Li Y, Edwards T.J, Kurniawan N.D, Richards L.J, Jiang T (2016). Altered structural connectome in adolescent socially isolated mice. Neuroimage.

[27] Schrempft S, Jackowska M, Hamer M, Steptoe A (2019). Associations between social isolation, loneliness, and objective physical activity in older men and women. BMC Public Health.

[28] Masi C.M, Chen H.Y, Hawkley L.C, Cacioppo J.T (2011). A meta-analysis of interventions to reduce loneliness. Pers. Soc. Psychol. Rev.

[29] Rai A.R, Madhyastha S, Prabhu L.V, Saralaya V.V, Sahu S.S, Rao G (2014). Resveratrol reverses the restraint stress-induced cognitive dysfunction involving brain antioxidant system in rats. Int. J. Pharm. Pharm. Sci.

[30] Sharifi-Rad M, Anil Kumar N.V, Zucca P, Varoni E.M, Dini L, Panzarini E, Rajkovic J, Tsouh Fokou P.V, Azzini E, Peluso I, Sharifi-Rad J (2020). Lifestyle, oxidative stress, and antioxidants:back and forth in the pathophysiology of chronic diseases. Front. Physiol.

[31] Amiri S, Amini-Khoei H, Haj-Mirzaian A, Rahimi-Balaei M, Naserzadeh P, Dehpour A, Mehr S.E, Hosseini M.J (2015). Tropisetron attenuated the anxiogenic effects of social isolation by modulating nitrergic system and mitochondrial function. Biochim. Biophys. Acta.

[32] Haj-Mirzaian A, Amiri S, Kordjazy N, Rahimi-Balaei M, Haj-Mirzaian A, Marzban H, Aminzadeh A, Dehpour A.R, Mehr S.E (2015). Blockade of NMDA receptors reverses the depressant, but not anxiogenic effect of adolescence social isolation in mice. Eur. J. Pharmacol.

[33] Amiri S, Haj-Mirzaian A, Amini-Khoei H, Momeny M, Shirzadian A, Rahimi-Balaei M, Dehpour A.R, Mehr S.E (2016). NMDA receptor antagonists attenuate the proconvulsant effect of juvenile social isolation in male mice. Brain Res. Bull.

[34] Zlatković J, Filipović D (2013). Chronic social isolation induces NF-kB activation and upregulation of iNOS protein expression in rat prefrontal cortex. Neurochem. Int.

[35] Workman J.L, Fonken L.K, Gusfa J, Kassouf K.M, Nelson R.J (2011). Post-weaning environmental enrichment alters affective responses and interacts with behavioral testing to alter nNOS immunoreactivity. Pharmacol. Biochem. Behav.

[36] Xiong Y, Hong H, Liu C, Zhang Y.Q (2023). Social isolation and the brain:Effects and mechanisms. Mol. Psychiatry.

[37] Tang W, Shin J.D, Jadhav S.P (2021). Multiple time-scales of decision-making in the hippocampus and prefrontal cortex. Elife.

[38] Jobson D.D, Hase Y, Clarkson A.N, Kalaria R.N (2021). The role of the medial prefrontal cortex in cognition, ageing and dementia. Brain Commun.

[39] Takehara-Nishiuchi K (2020). Prefrontal-hippocampal interaction during the encoding of new memories. Brain Neurosci. Adv.

[40] Sharpe M.J, Killcross S (2018). Modulation of attention and action in the medial prefrontal cortex of rats. Psychol. Rev.

[41] Woo E, Sansing L.H, Arnsten A.F, Datta D (2021). Chronic stress weakens connectivity in the prefrontal cortex:Architectural and molecular changes. Chronic Stress (Thousand Oaks).

[42] Takahashi A (2022). The role of social isolation stress in escalated aggression in rodent models. Neurosci. Res.

[43] Belleau E.L, Treadway M.T, Pizzagalli D.A (2019). The impact of stress and major depressive disorder on hippocampal and medial prefrontal cortex morphology. Biol. Psychiatry.

[44] Fenster R.J, Lebois L.A, Ressler K.J, Suh J (2018). Brain circuit dysfunction in post-traumatic stress disorder:From mouse to man. Nat. Rev. Neurosci.

[45] Sharma V, Sharma R, Gautam D.S, Kuca K, Nepovimova E, Martins N (2020). Role of Vacha (*Acorus calamus* Linn.) in neurological and metabolic disorders:Evidence from ethnopharmacology, phytochemistry, pharmacology and clinical study. J. Clin. Med.

[46] Oli B.S, Rauniyar A, Chad D (2021). A review on the significance of the medicinal plant *Acorus calamus*. Asian J. Pharmacogn.

[47] Das B.K, Swamy A.V, Koti B.C, Gadad P.C (2019). Experimental evidence for use of *Acorus calamus* (asarone) for cancer chemoprevention. Heliyon.

[48] Esfandiari E, Ghanadian M, Rashidi B, Mokhtarian A, Vatankhah A.M (2018). The effects of *Acorus calamus* L. in preventing memory loss, anxiety, and oxidative stress on lipopolysaccharide-induced neuroinflammation rat models. Int. J. Prev. Med.

[49] Saroya A.S, Singh J, Saroya A.S, Singh J (2018). Neuropharmacology of *Acorus calamus* L. In:Pharmacotherapeutic Potential of Natural Products in Neurological Disorders.

[50] Ukkirapandian K, Kayalvizhi E, Udaykumar K.P, Kandhi S, Muthulakshmi R, Elumalai K, Gandhi S, Rangasmy M (2022). The neuroprotective role *of Acorus calamus* in developmental and histopathological changes in autism-induced wistar rats. Cureus.

[51] Faheem M, Ameer S, Khan A.W, Haseeb M, Raza Q, Shah F.A, Khusro A, Aarti C, Sahibzada M.U.K, Batiha G.E.S, Koirala N (2022). A comprehensive review on antiepileptic properties of medicinal plants. Arab. J. Chem.

[52] Allahverdiyev O, Dzhafar S, Berköz M, Yıldırım M (2018). Advances in current medication and new therapeutic approaches in epilepsy. East. J. Med.

[53] Choudhary N, Singh V (2019). Insights about multi-targeting and synergistic neuromodulators in Ayurvedic herbs against epilepsy:Integrated computational studies on drug-target and protein-protein interaction networks. Sci. Rep.

[54] Sundaramahalingam M, Ramasundaram S, Rathinasamy S.D, Natarajan R.P, Somasundaram T (2013). Role of *Acorus calamus* and alpha-asarone on hippocampal dependent memory in noise stress exposed rats. Pak. J. Biol. Sci.

[55] Jiji K.N, Muralidharan P (2020). A comprehensive review on neuropharmacological potential of *Acorus calamus* linn. Plant Arch.

[56] Yousuf S, Haq S.M.U, Rasool A, Zulfajri M, Hanafiah M.M, Huang G.G, Mahboob M (2020). Evaluation of Antidepressant Activity of *Acorus calamus* L. Rizhome Extract in Mice. Research Square,North Carolina.

[57] Forouzanfar F, Hosseinzadeh H (2018). Medicinal herbs in the treatment of neuropathic pain:A review. Iran. J. Basic Med. Sci.

[58] Singh A.P (1999). Government of India notifies the rules for breeding of and conducting animal experiments. Indian J. Pharmacol.

[59] Bures J, Burešová O, Huston J.P (2016). Techniques and Basic Experiments for the Study of Brain and Behavior. Elsevier Science Publishers, Netherlands.

[60] Nandi A, Virmani G, Barve A, Marathe S (2021). Dbscorer:An open-source software for automated accurate analysis of rodent behavior in forced swim test and tail suspension test. eNeuro.

[61] Koracevic D, Koracevic G, Djordjevic V, Andrejevic S, Cosic V (2001). Method for the measurement of antioxidant activity in human fluids. J. Clin. Pathol.

[62] Marklund S, Marklund G (1974). Involvement of the superoxide anion radical in the autoxidation of pyrogallol and a convenient assay for superoxide dismutase. Eur. J. Biochem.

[63] Rao G.M, Rao A.V, Raja A, Rao S, Rao A (2000). Role of antioxidant enzymes in brain tumours. Clin. Chim. Acta.

[64] Lowry O.H, Rosebrough N.J, Farr A.L, Randall R.J (1951). Protein measurement with the Folin phenol reagent. Biol. Chem.

[65] Gorantla V. R, Thomas S.E, Millis R.M (2019). Environmental enrichment and brain neuroplasticity in the kainate rat model of temporal lobe epilepsy. J. Epilepsy Res.

[66] Grigoryan G.A, Pavlova I.V, Zaichenko M.I (2022). Effects of social isolation on the development of anxiety and depression-like behavior in model experiments in animals. Neurosci. Behav. Physiol.

[67] Zhang Y.F, Janke E, Bhattarai J.P, Wesson D.W, Ma M (2022). Self-directed orofacial grooming promotes social attraction in mice via chemosensory communication. iScience.

[68] Chaoui N, Anarghou H, Laaroussi M, Essaidi O, Najimi M, Chigr F (2022). Long lasting effect of acute restraint stress on behavior and brain anti-oxidative status. AIMS Neurosci.

[69] Rauniar G.P, Deo S, Bhattacharya S.K (2007). Evaluation of anxiolytic activity of tensarin in mice. Kathmandu Univ. Med. J. (KUMJ).

[70] Bhat S.D, Ashok B.K, Acharya R, Ravishankar B.A (2012). Comparative acute toxicity evaluation of raw and classically processed rhizomes of Vacha (*Acorus calamus* Linn.). Indian J. Nat. Prod. Resour.

[71] Pushpa V.H, Padmaja S.K, Suresha R.N, Vaibhavi P.S, Kalabharathi H.L, Satish A.M, Naidu S (2013). Antidepressant activity of methanolic extract of *Acorus calamus* leaves in albino mice. Int. J. Pharm. Tech.

[72] Patel S, Rajshree N.I.T.I, Shah P (2016). Evaluation of antidepressant activity of herbomineral formulation. Int. J. Pharm. Pharm. Sci.

[73] Becker M, Pinhasov A, Ornoy A (2021). Animal models of depression:What can they teach us about the human disease?. Diagnostics (Basel).

[74] Vrankova S, Galandakova Z, Benko J, Cebova M, Riecansky I, Pechanova O (2021). Duration of social isolation affects production of nitric oxide in the rat brain. Int. J. Mol. Sci.

[75] Shukla P.K, Khanna V.K, Ali M.M, Maurya R, Khan M.Y, Srimal R.C (2006). Neuroprotective effect of *Acorus calamus* against middle cerebral artery occlusion-induced ischaemia in rat. Hum. Exp. Toxicol.

